# Occlusion‐robust markerless surgical instrument pose estimation

**DOI:** 10.1049/htl2.12100

**Published:** 2024-11-27

**Authors:** Haozheng Xu, Stamatia Giannarou

**Affiliations:** ^1^ Hamlyn Centre for Robotic Surgery, Department of Surgery and Cancer Imperial College London London UK

**Keywords:** endoscopes, medical robotics, pose estimation

## Abstract

The estimation of the pose of surgical instruments is important in Robot‐assisted Minimally Invasive Surgery (RMIS) to assist surgical navigation and enable autonomous robotic task execution. The performance of current instrument pose estimation methods deteriorates significantly in the presence of partial tool visibility, occlusions, and changes in the surgical scene. In this work, a vision‐based framework is proposed for markerless estimation of the 6DoF pose of surgical instruments. To deal with partial instrument visibility, a keypoint object representation is used and stable and accurate instrument poses are computed using a PnP solver. To boost the learning process of the model under occlusion, a new mask‐based data augmentation approach has been proposed. To validate the model, a dataset for instrument pose estimation with highly accurate ground truth data has been generated using different surgical robotic instruments. The proposed network can achieve submillimeter accuracy and the experimental results verify its generalisability to different shapes of occlusion.

## Graphical abstract

This research addresses the challenge of estimating the pose of surgical instruments in Robot‐assisted Minimally Invasive Surgery (RMIS), which is crucial for surgical navigation and autonomous task execution. Existing methods struggle with accuracy when visibility is limited by occlusions or scene changes. To improve performance, the study introduces a vision‐based framework for markerless, 6DoF pose estimation of surgical instruments. It uses a keypoint‐based object representation and a PnP solver to accurately compute poses, even with partial visibility. A novel mask‐based data augmentation technique is proposed to enhance model learning under occlusions. It achieves submillimeter accuracy and demonstrates robust generalisability across different occlusion scenarios.

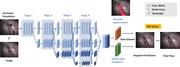

## 1 INTRODUCTION

Robot‐assisted minimally invasive surgery (RMIS) has evolved significantly in the last decades thanks to the advances in Artificial Intelligence (AI) and surgical robotics such as the da Vinci™platform, which provide surgical assistance through enhanced visualisation and feedback control. An important task in RMIS is the tracking of surgical tools. This involves the estimation of the 3D position and orientation of the tool as it moves with six degrees of freedom (6DoF).

In surgical tracking tasks, external hardware such as depth cameras, and optical and electromagnetic trackers have been widely used [1, 2]. These methods may require markers to be attached to surgical tools and introduce extra equipment to the operating theatre. However, this can be costly, and impractical and it requires additional hardware calibration and software installation. In contrast, vision‐based methods provide a practical and cost‐effective approach to tool tracking without requiring any modifications on the hardware setup or the attachment of external markers.

Early vision‐based methods for surgical tool tracking include marker‐based and markerless approaches. Since most of the surgical instruments including those of the Da Vinci™ surgical robotic system are cylindrical objects, emphasis has been given to the design of cylindrical markers [3, 4]. These patterns consist of an array of blobs and dots which are detected on the 2D image plane to estimate the 6DoF pose of the instrument. One limitation of these methods is that the marker needs to be kept in the camera's field of view (FoV). In addition, the attachment of external markers requires sterilization and calibration.

This has steered the research focus into the development of markerless methods based on computer vision algorithms. Most surgical instrument tracking methods [5, 6, 7] consist of two steps, (i) instrument segmentation and (ii) pose estimation. These methods first segment the 2D mask of the instrument on the image, then estimate the 3D pose given the prior knowledge of the 3D model and geometry primitives. However, these two‐stage methods make it difficult to accurately estimate the rotation along the central axis of instruments, since the region change on the image is insensitive to the axial rotation. These two‐stage methods rely on the accurate detection of image features like the tip or center line of the instrument, which is unstable in low‐light or high‐reflection scenarios due to endoscopic illumination.

Recently, some deep learning methods were proposed to directly estimate the 3D pose of the object in natural scenes but they have several challenges in surgical scenarios [8, 9, 10, 11, 12, 13]. The first challenge is the partial visibility of the instrument. The surgical camera needs to be very close to the instrument and tissue due to the limited operating space. In this case, only part of the instrument will be in the FOV which may affect the performance of pose estimation methods based on object detection. The second challenge is the occlusion of the tracked instrument. The surgical tool will frequently interact with organs and tissue, which can occlude the tip of the instrument. The visual features of the tip are vital for the pose estimation and occlusions can make the pose estimation unstable. Furthermore, the varying lighting conditions in the surgical environment and specular reflections on the tissue and the tool, affect significantly the appearance and texture of the instrument. Another challenge is the difficulty of acquiring vast and accurate training data. Although the pose of the instrument can be acquired from the kinematic information of a surgical robotic arm, the error of this estimated pose is in the range of several mm. Hence, robot kinematics can not be used to acquire ground truth tool poses and another reliable method needs to be developed to acquire a large amount of image and tool pose data.

In this work, we propose a vision‐based framework to estimate the 6DoF pose of surgical instruments without relying on external markers. To enable our method to efficiently deal with partial object visibility, a keypoint object representation is used. For this purpose, a keypoint prediction module is introduced to detect 2D keypoints on the shaft of the instrument. These keypoints correspond to 3D points sampled from the CAD model of the instrument. Stable and accurate object pose is computed using the PnP solver [14] based on the 2D‐3D correspondences. Our contributions are:

1. A feature backbone has been designed by adapting the HRNet to extract features at multiple resolutions and achieve high performance on high‐resolution endoscopic images.
2. A new mask‐based data augmentation approach has been proposed to increase the robustness of the pose estimation to partial instrument occlusion.
3. To validate our model, a dataset for 6DoF instrument pose estimation with highly accurate ground truth data has been generated using different surgical robotic instruments. The dataset will become publicly available upon publication of this work.

The proposed network can achieve sub‐millimeter accuracy. Also, our experimental results verify its generalisability and robustness to different shapes of occlusion.

## 2 METHODS

Our approach is designed to determine the 6DoF pose of a surgical instrument utilizing a single RGB image. Given an image I and a collection of n sparse 3D points ${\left\{z_{i}\right\}}_{i=1}^{n}$ located on the instrument, our initial step is projecting the sparse 3D points onto the image, generating a corresponding set of 2D keypoints ${\left\{x_{i}\right\}}_{i=1}^{n}$ onto the image. These 3D points are sampled from the instrument's CAD model by applying the farthest point sampling (FPS) algorithm [15]. Subsequently, the instrument's pose is derived through a RANSAC‐based Perspective‐n‐Point (PnP) methodology, which utilizes the 2D‐3D point correspondences for pose estimation. Moreover, our method attains resilience against partial occlusion of the instrument by implementing mask‐based data augmentation.

### 2.1 Keypoint prediction module

**FIGURE 1:**
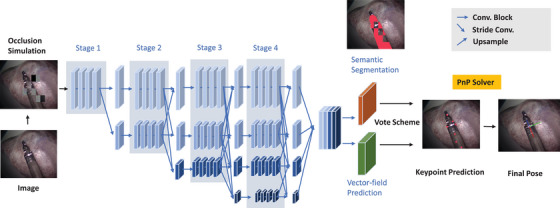
The overview of our proposed pose estimation network. RGB images labeled with semantic masks and 2D keypoint locations are used to train the model.

This module aims to identify on the instrument the 2D keypoints ${\left\{x_{i}\right\}}_{i=1}^{n}$. We propose a two‐stage pipeline. First, the keypoint prediction module predicts the 2D location of each keypoint, then the PnP algorithm is used to estimate the 6DoF pose given the known 3D location of each keypoint. For this purpose, high‐resolution feature maps which contain rich semantic and texture information are extracted by adapting the HRNet V2 model [16]. The HRNet is composed of multiple branches with different resolutions. This allows the network to extract both high‐level semantic representations and low‐level spatial features from the image data. The stage 4 outputs of HRNet V2 consist of 4 different scale feature maps representing high‐to‐low level image features. In the original HRNet V2 paper, only the largest feature map is used for segmentation. The other feature maps are discarded due to size incompatibility. In our framework, the 4 different scale feature maps are upsampled to the size of the largest map first. Then two 1x1 convolution kernels are added to fuse the feature maps to maintain high‐resolution image representations through the whole process. Our experiments have shown that combining the 4 different scale feature maps increases the model's performance. The concatenated feature map is fed to the instrument segmentation branch and the vector‐field prediction branch. These branches consist of 1×1 convolutions. The proposed model is shown in Figure 1.

Rather than directly regressing the 2D keypoint localization like [9], in this work a special format of unit vector maps [10] is used to represent the keypoint localization. It is based on the property of a rigid body where the relative position between different parts of a rigid body is fixed no matter how it translates or rotates. Once one part of the rigid body is visible, the rest of the rigid body parts can be inferred even under occlusion.

**FIGURE 2:**
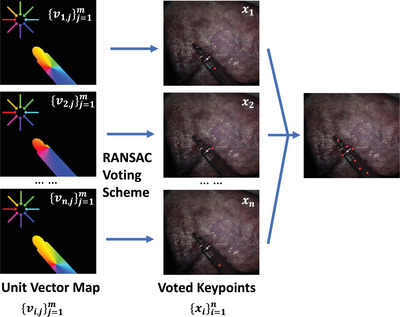
Illustration of unit vector map generation.

For an image containing M pixels, the semantic segmentation branch outputs the segmentation map ${\left\{{\mathrm{seg}}_{j}\right\}}_{j=1}^{M}$ and the vector‐field prediction branch outputs the unit vector maps ${\left\{v_{i,j}\right\}}_{i=1,j=1}^{n,M}$. To localize keypoints, the outputs from the two branches are combined to generate the filtered unit vector map ${\left\{v_{i,j}\right\}}_{i=1,j=1}^{n,m}$ where m<M. The filtered unit vector map is illustrated in Figure 2. The black pixels not belonging to the instrument are masked out by the segmentation branch while the colourful pixels belonging to the instrument are maintained. The colour of every pixel represents a unit vector towards a specific direction. Therefore, each map represents the object‐relevant unit vectors ${\left\{v_{i,j}\right\}}_{j=1}^{m}$ towards each keypoint $x_{i}$. A RANSAC‐based [17] voting scheme is then followed to generate candidate keypoint locations $\left\{h_{i,k}\right\}$. These locations are at the intersection of two random vectors in ${\left\{v_{j}\right\}}_{j=1}^{m}$.

Then, the weight for each intersection is estimated as:

(1) $$w_{i,k}=\sum \frac{{\left(h_{i,k}\;-p_{j}\right)}^{T}}{∥h_{i,k}\;-p_{j}∥}v_{j}\;,where\frac{{\left(h_{i,k}\;-p_{j}\right)}^{T}}{∥h_{i,k}\;-p_{j}∥}v_{j}\;\geq \theta .$$

Here, θ is a threshold that we set to 0.99. The keypoint $x_{i}$ is the weighted average of the intersections:

(2) $$x_{i}=\frac{\sum w_{i,k}h_{i,k}}{\sum w_{i,k}}.$$

The final keypoint location is the mean of the candidate keypoint locations. To train our model to predict keypoints on the instrument, a smooth L1 loss is used as below:

(3) $$Loss_{keypoints}=\frac{1}{N}\sum_{i}SmoothL_{1}\left(x_{i}-{\hat{x}}_{i}\right),$$

where $x_{i}$ and ${\hat{x}}_{i}$ are the predicted and ground truth keypoint predictions, respectively. The Smooth L1 loss is defined as:

(4) $$\text{Smooth L1}\left(x\right)=\left\{\begin{array}{cc} 0.5x^{2} & \text{if}\;|x|<1\\ |x|-0.5 & \text{otherwise} \end{array}\right..$$

The segmentation branch predicts whether a pixel belongs to the instrument or not via a binary cross‐entropy loss:

(5) $$Loss_{seg}=-\sum_{j=1}^{M}\left(\hat{seg_{j}}\log\left(seg_{j}\right)+\left(1-\hat{seg_{j}}\right)log\left(1-seg_{j}\right)\right),$$

where $seg_{j}$ and $\hat{seg_{j}}$ are the predicted and ground truth segmentation labels for pixel p, respectively.

### 2.2 Mask‐based data augmentation for occlusion

The task of pose estimation is significantly complicated by the partial occlusion of surgical instruments, as occlusion can obscure crucial visual features on the instrument's surface, such as edges, blobs, and corners. In light of this challenge, we introduce an innovative data augmentation technique in our study. This technique is designed to emulate various forms and degrees of instrument occlusions, along with intensity fluctuations, within our training dataset. The ultimate aim is to bolster the generalisability of our pose estimation model, ensuring its consistent performance in the face of diverse shapes and degrees of partial instrument occlusions.

Drawing inspiration from the hide‐and‐seek methodology [18] and the Masked Auto‐encoder (MAE) technique [19], the concept of randomly masking image patches has found extensive utilization in self‐supervised image reconstruction assignments. The act of masking segments of the image necessitates the network to decipher the geometric interplay between adjacent patches, thereby facilitating the reconstruction of the concealed image portion. These methods use masking to remove image areas to make the model focus on the visible parts of the image during learning. Our proposed mask‐based data augmentation technique is the first method developed for supervised instrument pose estimation. The aim of our method is to introduce occlusions to help the model distinguish the object‐relevant pixels from occlusions for accurate pose estimation under occlusion.

We advocate a strategy tailored for the generation of training data suitable for our pose estimation method. As part of each training epoch, an array of customary data augmentations, inclusive of translation, rotation, scaling, and colour jitter, are initially and randomly imposed upon the raw images and their corresponding segmentation masks in tandem.

**FIGURE 3:**
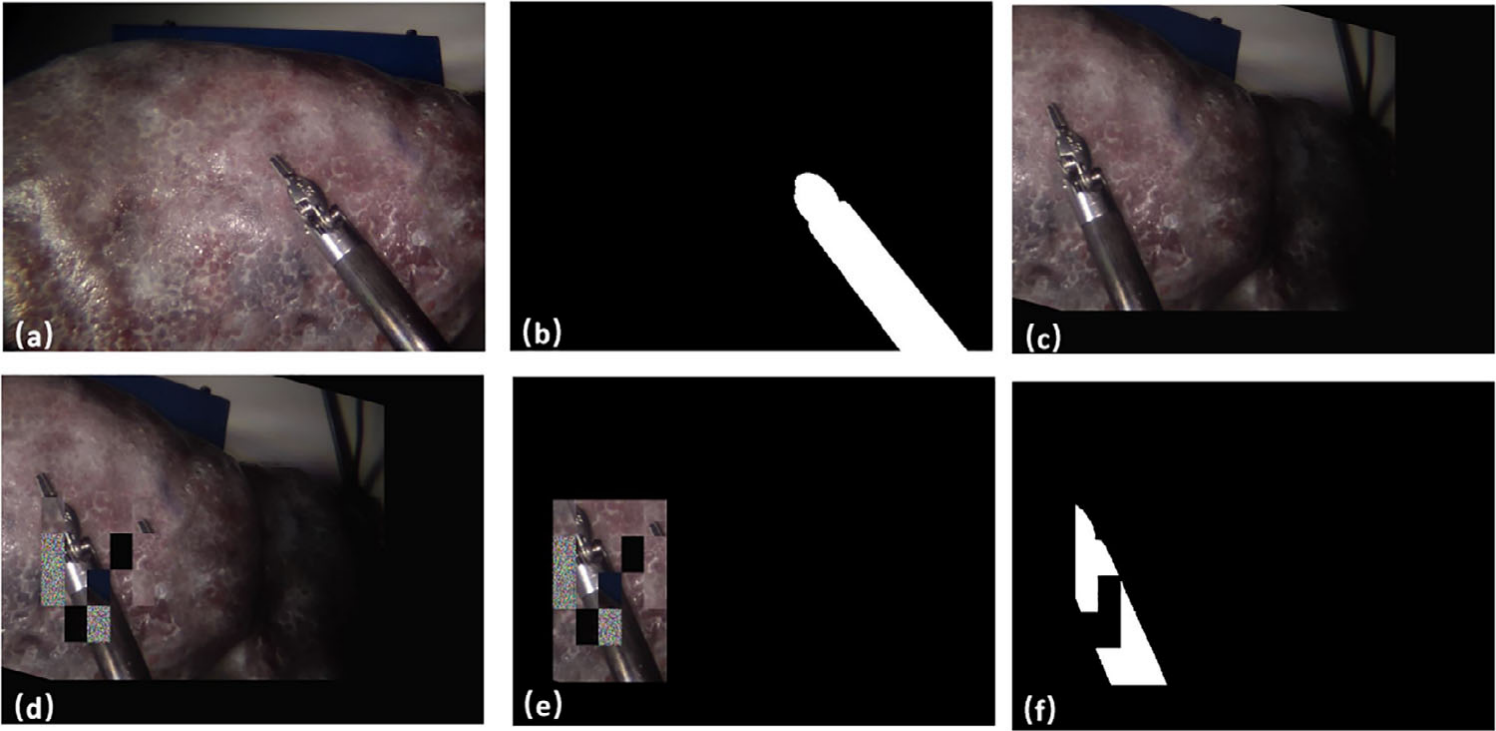
Data Augmentation Process. (a) represents the raw image. (b) Raw Segmentation Mask. (c) represents regular augmentation. (d) Patch Occlusions. (e) Mask out of the background. (f) Augmented Segmentation Mask.

A bounding box is formulated to encapsulate the entire instrument's body as seen in Figure 3e. The region within this bounding box is then subdivided into numerous grids, as illustrated in Figures 3c and 3f. Each grid is subject to substitution by either a noise patch or a shifted image patch. The percentage of grids that have been replaced by noise patches is determined by the probability $p_{occlusion}$. These noise patches are exclusively comprised of pixels with randomly assigned values ranging from 0 to 255, as depicted in Figure 3c. The shifted image patch is derived from an area outside the bounding box, randomly chosen from regions not containing the instrument. Moreover, for a subset of the training dataset, the entire background external to the bounding box is eliminated, as shown in Figure 3d, thereby mitigating the background's influence on network training. This strategic approach serves to elevate the model's adaptability to variations in the surgical environment, as well as to the form and degree of instrument occlusion. Given that our keypoint prediction module individually processes each pixel of the image, the partial visibility of the instrument exerts no detrimental impact on the learning process related to the instrument's pose.

Our proposed augmentation method efficiently simulates occlusion in surgery, not found in previous works. Existing masking methods apply fixed‐size masks to RGB images with 2D transformations. Our method simulates diverse shapes of occlusion using grid masks. It also transforms the image in 3D with rotation and translation, adds random noise and removes the background. More importantly, existing methods act only on the input, remove the masked image part and let the model recover this lost information. Our masking is applied to both the input and the ground truth (GT) segmentation and unit vector maps, aiming to add disturbance to the image then promote the model to discriminate between object and background, as well as estimate pose. Hence, in our augmentation, the mask is created and applied in a novel way compared to existing methods.

### 2.3 Generation of pose estimation datasets

In order to closely approximate real‐world applications, video data was captured using the stereo laparoscope of a da Vinci™Si surgical robot, employing a 30 degree, 8.5 mm Si Endoscope. Given that our methodology necessitates a monocular image as input, we solely utilized the left laparoscope camera. The original resolution of the footage is 1920×1080 pixels. However, considering the substantial memory demands associated with high‐resolution images, the final images utilized for deep learning training were downsampled by a factor of 2. The video capture procedure was programmed in Python and executed at a rate of 20 Hz on a computer outfitted with an Intel™Core (i7‐8700) CPU operating at 3.20 GHz and equipped with 16 GB of RAM.

Although the instrument pose can be acquired from the forwarded kinematic information from the machine arm, the kinematic information is not accurate enough to generate ground truth labels. In [7], the performance of kinematics‐based pose estimation methods has been compared to vision‐based methods and has been shown that the kinematics error is over 5 cm.

Instead, we 3D printed an attachment with a keydot pattern on the head. To get the ground truth pose between the camera coordinate and instrument coordinate $T_{I}^{C}$, we initially get $T_{KP}^{C}$ from the image. The transformation from instrument coordinates I to the keydot pattern coordinate KP can be represented as $T_{I}^{KP}$. Since the transformation $T_{I}^{KP}$ is constant and important to the ground truth labelling, we extract the geometric information of the attached holder and apply a manual adjustment to increase the transformation accuracy. Given the CAD model of the instrument, we generate the ground truth mask segmentation by 2D projection. In addition, we sample n = 10 3D keypoints ${\left\{z_{i}\right\}}_{i=1}^{n}$. At time t, the 2D keypoints ${\left\{x_{i}\right\}}_{i=1}^{n}$ can be projected on the image plane as:

(6) $$x_{i,t}=KT_{KP}^{C}T_{I}^{KP}z_{i,t},$$

where K is the camera calibration matrix.

Eventually, three datasets were collected:

1) Dataset I: This dataset contains videos captured using the Endowrist™Large Needle Driver, all of which are devoid of occlusion. The background for these videos is a high‐fidelity liver phantom. In an effort to introduce variation in terms of lighting conditions, we opted for different levels of light source intensity, specifically 40%, 70%, and 100%. Furthermore, an additional light source was incorporated, the intensity of which was varied throughout the entire recording process. Similarly, the position and orientation of the background phantom were not kept constant during the recording. Any frames that depicted less than 20% of the instrument's tip were excluded from the dataset. Consequently, Dataset I comprises a total of 5945 frames designated for training purposes, along with 1630 frames earmarked for testing.
2) Dataset II: It contains the videos captured using the Endowrist™Prograsp Forceps without occlusion. The same light source, background, and variations of the scene were applied as in Dataset I. Dataset II contains 4784 frames for training and 2010 frames for testing.
3) Dataset III: This dataset includes the videos captured using as an instrument the Endowrist™Large Needle Driver with partial instrument occlusion. The occlusion is caused due to the presence of another surgical tool. To test the generalisability of the method, various occlusion objects were added including surgical instruments, scissors, tweezers, and cylindrical sticks. This dataset is only used for testing and contains 1506 frames.

### 2.4 Marker inpainting

**FIGURE 4:**
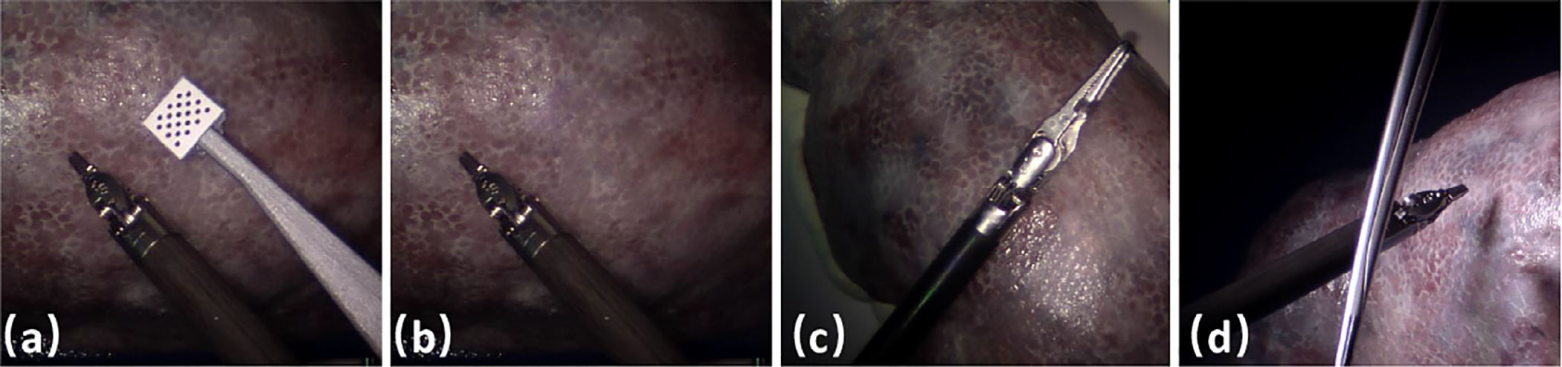
(a) Sample raw image from Dataset I. (b) The inpainting result of (a). (c) Sample image from Dataset II. (d) Sample image from Dataset III.

Given that the visible pattern could serve as prior knowledge for the training of the deep learning model, we employed an image inpainting model proposed by [20] to remove the marker. This is because the keydot marker would not be present in a real application and in our case is used only to generate ground truth data for our validation. A 2D mask of random size was projected onto the predetermined 3D pose of the marker, ensuring that the mask covers the marker to guarantee complete coverage. We used masks of random sizes to make sure the method cannot generate any consistent visual features as a shortcut that can be learned by any computer vision method. Simultaneously, the corner positions of the mask were allowed to vary within a specific range, ensuring that the mask differed for each frame, thereby preventing the introduction of any prior knowledge. As illustrated in Figure 4, the visible marker in the raw sub‐image (a) was effectively removed via inpainting, as depicted in sub‐image (b).

## 3 RESULTS AND EVALUATION

For our experiments, we utilized a workstation equipped with an Intel™Core i9‐12900K @5.20 GHz and an NVIDIA™RTX 3090 to train our neural network, with Dataset I and Dataset II serving as the training datasets. Meanwhile, Dataset III was employed to assess the model's robustness in the face of partial instrument occlusion, with an inference speed of approximately 30 fps recorded on this workstation.

Concerning our proposed mask‐based data augmentation technique, we opted to set the probability of mask occlusion within the range of 0.15 to 0.5, with the percentage of noise patch fixed at 0.4. Moreover, for each training sample, the probability of enacting mask occlusion and blackout was set at 0.6 and 0.2, respectively. An initial learning rate of $1\times 10^{-3}$ was selected, with a halving of this rate occurring every 20 epochs. The ADAM optimizer was employed with a momentum of 0.9.

To assess the performance of our pose estimation method, we evaluated our model in terms of the average 3D distance (ADD), the area under the curve (AUC curve of ADD), translation error, and rotation error. The ADD quantifies the mean distance between the points of the 3D tool model as transformed by the predicted and ground truth poses. To comprehensively evaluate the performance of the pose estimation method on a different scale, we generate the AUC curve of ADD, which illustrates the proportion of test samples where the ADD was less than the threshold plotted on the *x*‐axis, with higher thresholds indicative of greater accuracy. The inference frequency of our model on a workstation with RTX 3090 is 21 fps.

### 3.1 Performance without occlusion

**FIGURE 5:**
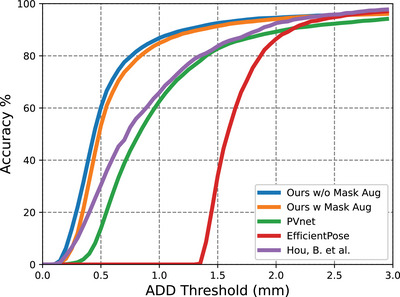
Accuracy‐threshold curve for test data in Dataset I.

**FIGURE 6:**
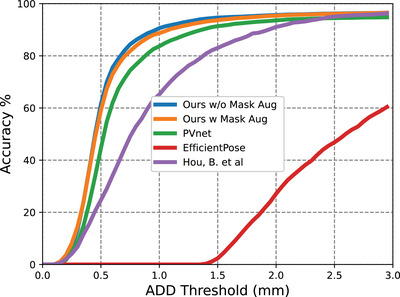
Accuracy‐threshold curve for test data in Dataset II.

**FIGURE 7:**
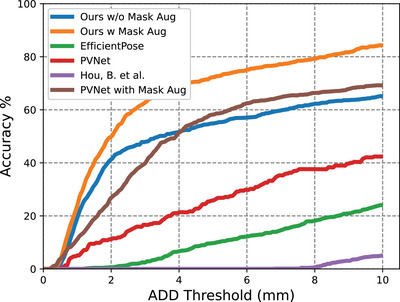
Accuracy‐threshold curve for test data in Dataset III.

The AUC curves of our method are displayed in Figures 5, 6 and 7. A comparison was made between PVNet [10], EfficientPose [21], Hou's work [13] and our method, with results shown in Table 1. Although EfficientPose performs well in the LineMOD benchmark [22], it yields the worst overall performance, struggling with rotation error due to its reliance on object‐level features. In contrast, Hou's model [13] achieves better accuracy by using direct pose estimation. In addition, PVNet also achieves high accuracy due to its keypoint representation detection, with our method outperforming both due to the use of HRNet as the backbone, which efficiently extracts feature maps from high‐resolution images. The multiple stages architecture of our method preserves rich semantic information and increases spatial accuracy. Additionally, the IoU of our segmentation for fully visible tools is 0.964 for the Large Needle Driver (LND) on Dataset I and 0.973 for the Prograsp Forceps (PG) on Dataset II. This means our model can accurately mask out the irrelevant pixels for further pose estimation.

**TABLE 1:** Results of our method on Dataset I and II.

	ADD (mm)		Translation error (mm)		Rotation error ( 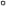 )	
Method	DS I	DS II	DS I	DS II	DS I	DS II
PVNet [10]	1.67	1.85	1.40	1.21	2.59	3.23
Hou, B. et al. [13]	1.45	1.04	1.01	1.20	1.20	1.39
EfficientPose [21]	5.19	10.22	1.28	4.24	149.21	129.75
Ours w/o Mask‐based Aug	0.92	0.72	0.91	0.84	**1.05**	1.22
Ours w Mask‐based Aug	**0.84**	**0.70**	**0.97**	**0.69**	1.21	**1.12**

### 3.2 Performance with occlusion

**FIGURE 8:**
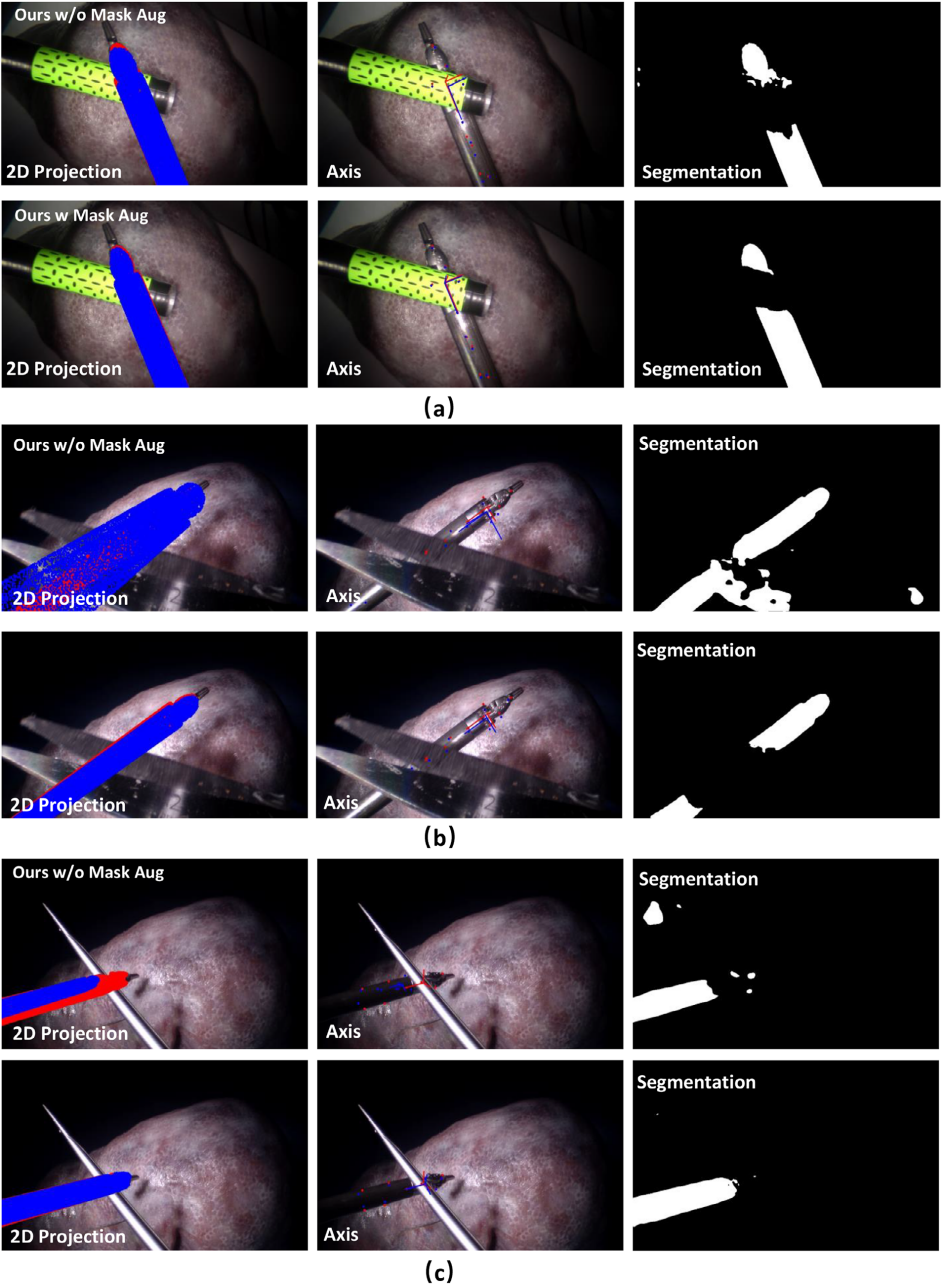
Occlusion example with (a) cylindrical objects, (b) scissors and (c) tweezers. Ground Truth data is labeled in red and predictions in blue.

During the collection of dataset III, we added partial occlusion over the instrument with multiple occlusion objects, as shown in Figure 4. Here we train our model purely on Dataset I for Endowrist™Large Needle Driver. We avoid any fine‐tuning on Dataset III to make sure the network has never learned the occlusion information before. We did an extra ablation study to analyze the effectiveness of the proposed mask‐based data augmentation methods on different models. As can be seen in Figure 7, there is a large accuracy drop for all methods on the occlusion task. Hou's model has the largest performance drop on pose estimation under occlusion since it learns the non‐linear pose directly from images which limits its generalisability, especially in the presence of occlusion. However, with the help of mask‐based data augmentation, the accuracy of our method can reach 60% when we set the ADD threshold to 4 mm, which means it can detect the instrument even under occlusion with significantly higher accuracy. This greatly illustrates the significance of our proposed mask‐based data augmentation method. As it can be seen in Figure 8, although the model has been trained with masked patches of rectangular shape, it can still recover the pose of the cylindrical instrument under partial occlusion of different shapes, without any prior knowledge. This verifies the robustness and generalisability of our model to the shape of the occlusion.

## 4 CONCLUSION

In this article, we proposed a keypoint prediction‐based pose estimation method for surgical instruments. An innovative mask‐based data augmentation method is designed to increase the robustness of the method to various occlusions which are common but challenging in surgical scenarios. To validate our method, a high‐quality dataset for surgical instruments is generated. Our proposed method can achieve submillimeter accuracy and our experiments verify the high generalisability and robustness of our model to different shapes of occlusion. So far, our proposed method can estimate the pose of rigid parts on objects such as surgical instruments including, imaging probes (ultrasound, gamma probes etc.), scalpels and da Vinci instruments. Our future work will focus on extending our method to estimate the pose of objects with rigidly‐deforming parts. Every rigid part of the tool can be considered as a separate object. Our proposed pose estimation model can be applied to each rigid part separately. In addition, our vision‐based method can be combined with kinematic data for higher accuracy and generalisabilty. For example, kinematic information can help alleviate any ambiguity regarding the rotation of the instrument along its axis.

## AUTHOR CONTRIBUTIONS

**Haozheng Xu**: Conceptualization; data curation; formal analysis; investigation; methodology; project administration; software; validation; visualization; writing—original draft; writing—review and editing. **Stamatia Giannarou**: Conceptualization; formal analysis; funding acquisition; methodology; resources; supervision; validation; writing—review and editing.

## CONFLICT OF INTEREST STATEMENT

The authors declare no conflicts of interest.

## Data Availability

The data that support the findings of this study are available from the corresponding author upon reasonable request.
